# *Trypanosoma cruzi* amastigotes that persist in the colon during chronic stage murine infections have a reduced replication rate

**DOI:** 10.1098/rsob.200261

**Published:** 2020-12-16

**Authors:** Alexander I. Ward, Francisco Olmo, Richard L. Atherton, Martin C. Taylor, John M. Kelly

**Affiliations:** Department of Infection Biology, London School of Hygiene and Tropical Medicine, London, UK

**Keywords:** *Trypanosoma cruzi*, chronic infection, dormancy, proliferation, replication

## Abstract

Chronic *Trypanosoma cruzi* infections are typically lifelong, with small numbers of parasites surviving in restricted tissue sites, which include the gastrointestinal tract. There is considerable debate about the replicative status of these persistent parasites and whether there is a role for dormancy in long-term infection. Here, we investigated *T. cruzi* proliferation in the colon of chronically infected mice using 5-ethynyl-2′deoxyuridine incorporation into DNA to provide ‘snapshots’ of parasite replication status. Highly sensitive imaging of the extremely rare infection foci, at single-cell resolution, revealed that parasites are three times more likely to be in S-phase during the acute stage than during the chronic stage. By implication, chronic infections of the colon are associated with a reduced rate of parasite replication. Despite this, very few host cells survived infection for more than 14 days, suggesting that *T. cruzi* persistence continues to involve regular cycles of replication, host cell lysis and re-infection. We could find no evidence for wide-spread dormancy in parasites that persist in this tissue reservoir.

## 1. Introduction

Disease latency, mediated by a wide range of mechanisms, is a common feature of viral, bacterial and parasitic infections [1–3]. However, there can be long-term consequences for the host, which include relapse and/or inflammatory pathology. The terms ‘persistent’, ‘dormant’ and ‘metabolically quiescent’ are used, often interchangeably, to describe pathogens in this state. The phenomenon has evolved independently and frequently in different pathogen groups, presumably because it acts to enhance survival and transmission. The ‘persister’ phenotype does not involve the acquisition of selected mutations, and is often associated with treatment failure, antibiotic tolerance being the best studied example [4,5]. In the case of Chagas disease, some form of dormancy or restricted replication has been widely postulated as a mechanism that might explain long-term parasite survival and the high rate of treatment failure [6].

Chagas disease is caused by the protozoan parasite *Trypanosoma cruzi*, which infects 6–7 million people, mainly in Latin America. Better drugs and innovative immunological interventions are urgently required. Human infection is normally initiated when faeces of the triatomine insect vector, contaminated with metacyclic trypomastigote forms of the parasite, come into contact with the bite wound, or when they are rubbed into mucous membranes. An acute parasitaemia develops, which can be asymptomatic, or manifest as generalized symptoms such as fever, headache and muscle pain. Suppression of the infection is then mediated by a CD8+ T-cell-mediated response which reduces parasite numbers to extremely low levels [7,8]. A subset of infected individuals (approx. 30%) eventually develop the classical Chagasic cardiac and/or digestive symptoms, although this can be decades after the acute stage infection. Dilated cardiac myopathy and digestive megasyndromes are the most common morbidities, and can often be fatal [9,10]. It remains to be established how the parasite is able to persist long term, albeit at very low levels, in the face of a robust adaptive immune response [11]. Furthermore, the reasons why treatment failures are a common outcome needs to be better understood at a mechanistic level to guide the design of improved chemotherapy [12]. In this context, the recent report of a non-proliferative form of *T. cruzi* that is refractory to treatment with the front-line drug benznidazole [13] could have important implications.

The ability of human parasites to enter a long-term quiescent state, in which both replication and metabolism are slowed, has been described in *Toxoplasma gondii* (the bradyzoite) [14] and some *Plasmodium* species [3]. As with many prokaryotic pathogens [15,16], the ‘dormant’ state involves lower levels of DNA synthesis and transcription, downregulation of energy catabolism and activation of DNA damage/cellular stress responses. In *T. gondii*, a master transcription factor (BFD1), activated by stress response pathways, initiates the onset of bradyzoite development [17]. The precise triggers that lead to differentiation into the quiescent hypnozoite liver stage in some *Plasmodium* spp. have been elusive [3]. Among eukaryotic pathogens, these examples represent one end of the ‘dormancy spectrum’, in which entry into a quiescent metabolic state is for extended periods. It has also been tentatively proposed that *Leishmania donovani* can enter a form of dormancy, although the mechanisms involved are unknown [18]. The situation in other *Leishmania* spp. is more definitive, with the identification of quiescent intracellular amastigotes which exhibit a slower metabolic flux and a reduced replication rate [19,20]. This stops short of full long-term dormancy in which parasites enter G0/G1 cell cycle arrest. *Plasmodium falciparum* blood stage schizonts can also enter a transient state of dormancy, induced by treatment with the front-line drug artemisinin [21,22]. This capacity to respond to stress by halting progress through the cell cycle exists in most cells that have DNA damage sensing machinery [23,24]. The existence of a dormant phenotype in the African trypanosome, *Trypanosoma brucei*, beyond the G0/G1 arrested stumpy form required for onward transmission [25] remains speculative.

Observations of both *in vitro* and *in vivo T. cruzi* infections identified a subpopulation of non-dividing intracellular amastigotes that retained the ability to differentiate into flagellated trypomastigotes, which were then able to propagate the infection [13]. This phenomenon was defined as spontaneous dormancy on the basis of experiments that involved monitoring incorporation of the thymidine analogue 5-ethynyl-2′deoxyuridine (EdU) into replicating DNA, and use of the tracker dye CellTrace Violet (CTV) to mark non-dividing parasites. Whether this represents long-term metabolic quiescence analogous to that in *T. gondii* and *Plasmodium* spp*.*, a slow-replicating phenotype as in *Leishmania* spp*.*, or temporary arrest induced by stress, as exhibited by *P. falciparum* and all non-tumorous mammalian cells, is unresolved. In this latter context, the report that *T. cruzi* amastigotes have an intrinsic ability to reduce their replication rate by temporary cell cycle arrest in G1, as a response to stress, nutrient availability and drug treatment, may be of relevance [26]. It is not known whether these represent overlapping or distinct mechanisms for entering a quiescent state. This could have implications for drug design, immunological interventions and our understanding of *T. cruzi* persistence.

Using highly sensitive bioluminescence and fluorescence imaging [27–29], we demonstrated that the gastrointestinal tract, specifically the colon and stomach, is a key site of *T. cruzi* persistence during chronic murine infections. Smooth muscle myocytes in the circular muscle layer of the colonic gut wall are the predominant host cell type. In the chronic stage, the entire colon typically contains only a few hundred parasites, often concentrated in a small number of cells that can contain greater than 100 parasites. During the acute stage, however, when the parasite burden is considerably higher and many cells are infected, host cells containing greater than 50 parasites are rarely found. Persistent parasites are also frequently detected in the skin during chronic infections, and in C3H/HeN mice, the skeletal muscle [29,30]. Further studies have also shown that parasite replication is asynchronous in individual host cells, a process that is independent of tissue location or disease stage, that replication of the nuclear and mitochondrial genomes is non-coordinated within the intracellular population and that replicating amastigotes and non-replicating trypomastigotes can coexist in the same cell [31].

We have developed tissue processing protocols and imaging procedures that allow us to routinely detect *T. cruzi* persistence foci during chronic murine infections at single-cell resolution [28,29]. Here, we describe experiments which provide new insights into parasite persistence, and indicate that chronic infections are associated with a reduced rate of parasite replication.

## 2. Methods

### 2.1. Parasites, mice and cell lines

The *T. cruzi* bioluminescent:fluorescent lines CL-Luc::mNeon or CL-Luc::Scarlet [28] were used throughout. Epimastigotes were grown in RPMI-1640, supplemented with 10% fetal bovine serum (FBS, BioSera), haemin (17 µg ml^−1^), trypticase (4.2 mg ml^−1^), penicillin (100 U ml^−1^) and streptomycin (100 µg ml^−1^), at 28°C. Metacyclic trypomastigotes were generated by culturing epimastigotes to stationary phase. *In vitro* studies were performed with the MA104 and Vero African green monkey kidney cell lines. *In vivo* experiments were carried out using female C3H/HeN mice, initially aged 8–12 weeks, purchased from Charles River (UK). Mice were maintained under specific pathogen-free conditions in individually ventilated cages, with a 12 h light/dark cycle. They had access to food and water ad libitum.

### 2.2. CellTrace Violet *in vitro* assay

*Trypanosoma cruzi* trypomastigotes were isolated by centrifugation and allowed to recover for 2 h at 37°C in high-glucose DMEM medium with 10% FBS, and then labelled with CTV fluorescent dye (Thermo Fisher Scientific), according to the manufacturer's protocol. Briefly, 2 × 10^6^ trypomastigotes were washed in PBS and then incubated for 20 min at 37°C in 10, 5, 2 or 1 µM CTV, protected from light. Unbound dye was quenched by the addition of one volume FBS and incubating for 5 min at 37°C. After washing (×2) in fresh complete medium, trypomastigotes were used for infection. Vero cells maintained in RPMI 10% FBS were trypsinized and seeded at 10^4^ or 10^5^ cells per well in 24-well plates containing coverslips, or in eight-well Ibidi µ-slides, and allowed to attach for 6 h before infection. Trypomastigotes were added at a multiplicity of infection (MOI) of 10 : 1 (parasite : host cell) and allowed to invade overnight (16–18 h). Cultures were then washed with PBS (×3) to remove non-invading parasites, and infected cultures incubated in RPMI with 2% FCS. Coverslips were fixed at different timepoints by transfer into a plate containing 4% paraformaldehyde for 30 min, then stained and mounted for microscopy using Vectashield^®^ with DAPI, or with propidium iodide following RNase treatment.

Images and videos were acquired using an inverted Nikon Eclipse microscope. The slide containing the infected cells was moved along the *x*–*y* plane through a 580 nm LED illumination. Images and videos were collected using a 16-bit, 1-megapixel Pike AVT (F-100B) CCD camera set in the detector plane. An Olympus LMPlanFLN 40x/1.20 objective was used to collect the exit wave leaving the specimen. Time-lapse imaging was performed by placing the chamber slide on the microscope surrounded by an environmental chamber (OKOLab cage incubator) maintaining the cells and the microscope at 37°C and 5% CO_2_. Video projections and Z-stack sequences were created using the deconvolution app in the Nikon imaging software.

### 2.3. *In vitro* parasite culturing and EdU labelling

Tissue culture trypomastigotes (TCTs) were derived after infecting MA104 cells with metacyclic trypomastigotes. MA104 cells were cultured in Minimum Essential Medium Eagle (MEM, Sigma-Aldrich), supplemented with 5% FBS at 37°C, in 5% CO_2_. Twenty-four-well plates containing coverslips were seeded with 10^5^ cells per well and left for 48 h. After reaching 95–100% confluency, they were infected with TCTs at an MOI of 5 : 1 (parasite : host cell). Eighteen hours later, external parasites were removed by washing (×3), fresh supplemented MEM was added and the infections allowed to proceed.

EdU (Sigma-Aldrich) in PBS was diluted to the appropriate concentration in supplemented MEM. The medium was removed, and the infected monolayer washed (×2), and fresh medium including EdU was added. After the appropriate incubation period, cells were washed (×3). For EdU toxicity studies, parasite growth in infected cells was assessed in 96-well plates, 3 days after EdU addition, by measuring mNeonGreen fluorescence in a FLUOstar Omega plate reader (BMG LABTECH). Background fluorescence was calculated using uninfected MA104 cells (*n* = 6). For microscopy, cells in the 24-well plates were washed (×2) and incubated for 45 min in 4% paraformaldehyde diluted in PBS. Coverslips were then removed and washed (×2) in PBS. EdU incorporation was assessed using a Click-iT Plus EdU AlexaFluor 555 Imaging kit (Invitrogen), as per the manufacturer's instructions, followed by washing (×2) with PBS, with coverslips then mounted in Vectashield. To allow precise counting of amastigotes, cells were imaged in three dimensions with a Zeiss LSM880 confocal microscope, using the Image Browser overlay function to add scale bars. Images were exported as TIF files to generate figures.

### 2.4. *In vivo* infections

CB17 SCID mice were infected with 1 × 10^4^ *T. cruzi* CL-Luc::mNeon TCTs and monitored by bioluminescence imaging [32]. At the peak of infection (approx. 18 days), when bloodstream trypomastigotes were visible by microscopy, the mouse was euthanized [33] and infected blood obtained by exsanguination. Trypomastigotes were washed in Dulbecco's modified Eagle's medium, diluted to 5 × 10^3^ ml^−1^, and CH3/HeN mice injected i.p. with 1 × 10^3^ trypomastigotes.

### 2.5. *In vivo* EdU labelling

The standard 1-day EdU treatment involved two i.p. injections (12.5 mg kg^−1^ EdU in PBS) delivered 6 h apart. The second injection took place 18 h prior to sacrifice. For the 3.5-day treatment, the daily injection protocol (above) was extended for 3 days, with a final single injection on day 4, followed 4 h later with euthanization and necropsy. For acute stage experiments, mice were 14–16 days post-infection when EdU was administered, and for the chronic stage, mice had been infected for greater than 100 days. Organs and tissues were subjected to *ex vivo* imaging, bioluminescent foci from skeletal muscle and the colon were excised and processed for histology [33]. Where indicated, whole colons were removed from the gastrointestinal tract, split longitudinally, pinned luminal side up and the mucosal layer removed. Whole mounting of the entire external colonic gut wall was performed as described previously [29]. Parasites were identified by mNeonGreen fluorescence using confocal microscopy, and carefully removed, together with approximately 5 mm^2^ of surrounding tissue. Prior to a second mounting, tissue pieces were processed for EdU detection in accordance with the manufacturer's instructions [31]. Several tissue segments could be developed for visualization using 500 µl of Click-iT reaction mix.

### 2.6. Statistics

All statistical analyses were performed in GraphPad PRISM v. 8.0 and STATA v. 16.0., and the data expressed as the mean ± standard deviation of mean (s.d.), unless otherwise stated. *In vitro* EdU toxicity was calculated as % growth relative to non-treated controls. The data were fitted with a sigmoidal function with variable slope and the absolute IC_50_ value calculated by solving the function for *X* when *Y* = 50%. All data were tested for normality and homogeneity of variance using Shapiro–Wilk's and Levene's tests, respectively. Statistical comparisons between samples to analyse *in situ* EdU incorporation were performed using one-way ANOVA with *post hoc* Tukey's test for multiple comparisons. Datasets were analysed by non-parametric tests when variances were not homogeneous. The % EdU incorporation in colon tissue sections versus whole mount were compared using the Wilcoxon signed-rank test. The Kruskal–Wallis test was performed on the data in figure 6. Statistical significance was accepted where *p* ≤ 0.05 (*p ≤ 0.05, **p ≤ 0.01, ***p ≤ 0.001, ****p ≤ 0.0001).

## 3. Results

### 3.1. The CellTrace Violet tracker dye inhibits *T. cruzi* proliferation

**Figure 1.**
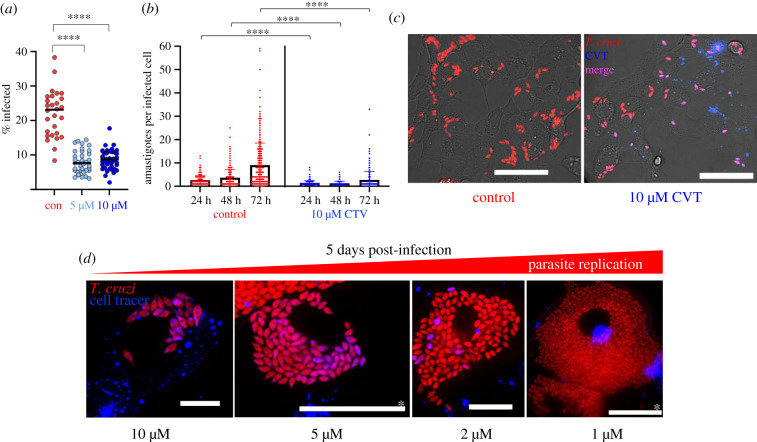
CTV reduces *T. cruzi* infectivity and inhibits intracellular proliferation. (*a*) *Trypanosoma cruzi* CLBr-Luc::Scarlet trypomastigotes were incubated with either 5 or 10 µM CTV for 20 min and used to infect Vero cell monolayers at an MOI of 10 : 1 (Methods). Eighteen hours later, infection efficiency was determined by inspecting a total of 2203 (control), 3781 (5 µM) and 3840 (10 µM) Vero cells (greater than 300 infected cells in each case). Each data point corresponds a randomly acquired image and represents the mean percentage of cells infected. Differences between columns were analysed using a parametric one-way ANOVA with Tukey's *post hoc* pair-wise comparisons. ****p ≤ 0.0001. (*b*) Vero cells infected with CTV−ve or CTV+ve trypomastigotes (as above) were incubated for the time periods indicated. The numbers of amastigotes per infected cell were then determined by analysing greater than 300 infected cells per treatment. Error bars represent the standard deviation from the mean. Data were analysed using a Wilcoxon rank-sum test. (*c*) Images of Vero cells 36 h after infection with CTV−ve (control) or CTV+ve trypomastigotes. Red, fluorescent *T. cruzi* amastigotes. Fluorescent parasites containing the CTV tracer dye appear as purple on a red fluorescent background. Size bars, 20 µm. (*d*) Images of Vero cells 5 days after infection with trypomastigotes that had been incubated with various concentrations of CTV, as indicated. Blue, intracellular vesicles containing CTV. See also electronic supplementary material, video S1. Size bars, 20 µm; those with * = 50 µm.

We sought to explore parasite replication by using CTV, a tracker dye that has been employed as a marker for spontaneous dormancy in *T. cruzi* amastigotes [13]. This succinimidyl ester dye diffuses into cells, binds covalently to the amine groups of proteins and becomes fluorescent following cleavage by intracellular esterases [34]. CTV fluorescence undergoes serial dilution with each round of parasite cell division, resulting in an inverse correlation between dye retention and proliferation rate. However, reports that CTV itself can inhibit cell division [35] prompted us to first investigate toxicity towards *T. cruzi.* Trypomastigotes were labelled by incubation for 20 min in 5 or 10 µM CTV (Methods), conditions that had been used previously to monitor parasite proliferation [13]. When these parasites were added to the Vero cell monolayer, we found they were 60% less infectious than trypomastigotes that had been incubated in the DMSO solvent alone (figure 1*a*). In the first 48 h post-infection, there was then limited division of intracellular CTV+ve parasites, with most trypanosomes in a state of growth arrest (figure 1*b,c*). By 72 h, replication had been more widely initiated, although the average number of amastigotes per infected cell was still significantly below that of the controls (figure 1*b*). Microscopy also revealed extensive heterogeneity in the intensity of CTV staining within the *T. cruzi* population, with many parasites failing to replicate, particularly in the first 36–48 h post-infection. At lower CTV concentrations (1 and 2 µM), growth inhibition was less evident and fewer parasites retained the dye at 5 days post-infection (figure 1*d*). Collectively, these experiments indicate that CTV is an inhibitor of trypomastigote infectivity and amastigote replication, and that the use of dye retention as a marker for dormancy and cell cycle arrest could lead to ambiguity. Furthermore, the heterogeneous nature of *T. cruzi* CTV staining, even within individual host cells (figure 1*c,d*), could result in differential growth and development rates within the same intracellular parasite population. Prior to infection, we removed non-bound CTV by quenching with addition of bovine serum (Methods). Despite this, some dye was taken up by mammalian cells and retained in stained vesicles for several days (figure 1*d*; electronic supplementary material, figure S1 and video S1). It was important to ensure co-localization of blue (CTV), red (fluorescent parasites) and/or green (DAPI-DNA) staining to avoid the risk of confusing amastigotes and spherical CTV-containing vesicles, both of which appear motile in the highly dynamic cytoplasmic environment.

### 3.2. Continuous EdU exposure can inhibit amastigote replication *in vitro*

**Figure 2.**
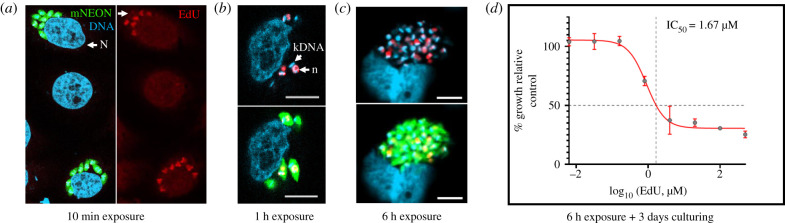
EdU incorporation by *T. cruzi* amastigotes *in vitro* is rapid, heterogeneous within the population and can inhibit parasite growth. (*a*) *Trypanosoma cruzi* CL-Luc::Neon trypomastigotes were used to infect MA104 cells at an MOI of 5 : 1 (Methods). Two days later, cultures were exposed to EdU (50 µM) for 10 min, and then examined by confocal microscopy. The image shows adjacent infected cells where in one instance, all eight amastigotes are EdU+ve, whereas in the other, 2/8 are EdU−ve (indicated by white arrowhead). *N*, host cell nucleus. (*b*) EdU labelling of amastigotes after 1 h exposure (40 µM). kDNA, kinetoplast DNA; *n*, parasite nucleus. (*c*) EdU labelling of amastigotes after 6 h exposure (10 µM). Scale bars, 10 µm in all cases. (*d*) MA104 cells were infected with trypomastigotes, and 2 days later, the cultures were exposed for 6 h to EdU at a range of concentrations, and then washed thoroughly. After a further 3 days incubation, amastigote growth was determined by assessing expression of the mNeonGreen reporter (Methods). The inhibition curve was plotted using GraphPad Prism to establish the concentration of EdU that conferred 50% growth inhibition compared to untreated controls. Data were derived from five replicates (CI_95_ 0.98 µM–4.83 µM).

We then used the thymidine analogue EdU to monitor parasite proliferation. In *T. cruzi*, incorporation of EdU provides a readout on the replicative status of both nuclear and mitochondrial DNA (kDNA) [28,31]. However, the procedure has to be used with caution, since EdU exposure *in vitro* can be associated with toxicity. In cultured mammalian cells, this is characterized by genome instability, DNA damage and cell cycle arrest [36–38]. Short-term exposure at lower concentrations (less than 12 h, less than 10 µM) appears to have less impact, and does not perturb cell cycle kinetics [39]. Toxicity against *T. cruzi in vitro* has also been shown to be dependent on exposure time; 4 h had only minor inhibitory effects on intracellular amastigotes, even at 70 µM, whereas with 24 h continuous exposure, the IC_50_ dropped to 70 nM [40]. By contrast, when infected cells were cultured for 72 h in the presence of 100 µM EdU, there was no reported inhibition of amastigote replication [13]. Given these conflicting observations, as a preliminary to *in vivo* studies, we assessed the *in vitro* kinetics and growth inhibitory effects of EdU on intracellular amastigotes of *T. cruzi* CL-Luc::Neon (a derivative of the CL Brener strain). This parasite reporter line expresses a fusion protein that is both bioluminescent and fluorescent [28]. After only 10 min exposure, amastigotes were clearly labelled, and it was possible to distinguish those that were EdU+ve from those that were EdU−ve (figure 2*a*). Similar heterogeneity, including differential labelling of nuclear and kDNA, was observed when infected cells were labelled for 1 or 6 h at different EdU concentrations (figure 2*b,c*). This pattern results from asynchronous amastigote replication [31], with EdU negativity/positivity determined by the position of individual parasites within the cell cycle during the period of exposure. When we assessed the extent of EdU growth inhibition after 6 h exposure, washing and examination 3 days later, we established an IC_50_ of 1.67 µM, although the level of inhibition plateaued at 70% (figure 2*d*). These outcomes are, therefore, consistent with those reported by Sykes *et al*. [40].

### 3.3. Reduced numbers of *T. cruzi* in S-phase during chronic stage of infections

**Figure 3.**
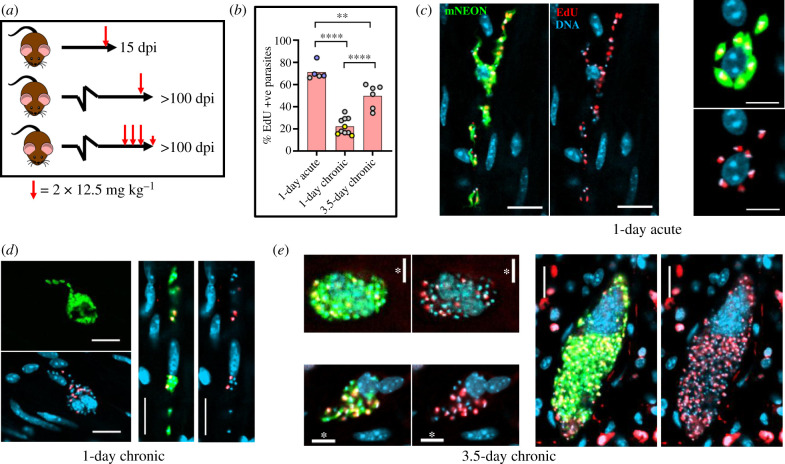
Intracellular *T. cruzi* replication, as inferred by EdU incorporation, is slower in the chronic stage than during acute infections. (*a*) Schematic showing experimental outline. C3H/HeN mice infected with *T. cruzi* CL-Luc::Neon were injected with EdU as indicated during the acute (15 days post-infection; dpi) or chronic stage (greater than 100 dpi). Each larger red arrow indicates 2 i.p. injections separated by 6 h. (*b*) Percentage of parasites that were EdU+ve under each treatment protocol. Colonic tissue was extracted from mice 18 h after the second injection (1-day treatment) or 4 h after the final injection (3.5 days treatment), and infected cells detected by *ex vivo* imaging and confocal microscopy (Methods) (electronic supplementary material, figure S2 and table S1) [29]. Each data point represents a single mouse. Blue data points indicate mice aged approximately 150 days at the start of acute infection, and act as age-matched controls. In the chronically infected mice, yellow data points indicate colons processed by standard histological sectioning, and grey points highlight those processed through peeling away of the mucosal layer and whole mounting the remaining colonic gut wall (Methods). No significant differences were observed in the % EdU+ve parasites in colonic sections processed by each method (Wilcoxon rank-sum test). For comparison of treatment conditions, statistical analysis was performed as described (Methods); ****p ≤ 0.0001 and **p ≤ 0.01. (*c*) Representative images of infected colonic muscle cells from an acute stage mouse. Labelling: parasites, green; DNA, blue (DAPI staining); EdU, red. EdU labelling on a green background appears yellow. (*d*,*e*) Images of infected colonic muscle tissue from chronically infected mice after EdU labelling using the 1-day and 3.5-day protocols, respectively (see also electronic supplementary material, figure S3). Scale bars, 20 µm, except where indicated; *10 µm.

We next compared the dynamics of EdU incorporation by parasites during acute and chronic murine infections with the *T. cruzi* CL-Luc::Neon strain. The elimination half-life (*T*_1/2_) of EdU in mice has not been determined, but with other thymidine analogues, the period is relatively short. For example, the bioavailability of bromodeoxyuridine (BrdU), a thymidine analogue also used in DNA labelling experiments, is less than 15 min [41]. Infected C3H/HeN mice were, therefore, given two EdU injections (each of 12.5 mg kg^−1^), 6 h apart (figure 3*a*), in an attempt to highlight a greater number of parasites where DNA replication was underway. At any one time, as judged by *in vitro* experiments, approximately 25–30% of amastigotes will be in S-phase [26]. In the majority of cases, the external gut wall mount methodology [29] was used to process the resulting tissue samples. The protocol enables the muscular coat, including the longitudinal and circular smooth muscle layers, which contain the majority of colon-localized parasites during chronic stage infections, to be visualized in their entirety at a three-dimensional level with single-cell resolution (Methods). Intracellular parasite numbers can be determined with accuracy by confocal microscopy using serial Z-stacking (electronic supplementary material, figure S2). On occasions, infected host cells in colonic tissue were also investigated by coupling *ex vivo* bioluminescence-guided excision and confocal microscopy (Methods) [31].

**Figure 4.**
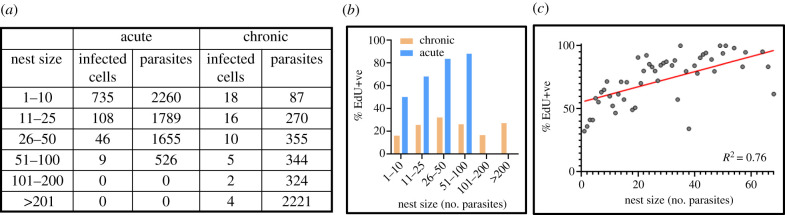
The inferred parasite replication rate is higher during the acute stage and correlates positively with nest size at this phase of the infection. (*a*) The number of infected cells (nests) detected in colonic gut wall tissue from mice in the acute (*n* = 5) and chronic (*n* = 7) stage, and the number of parasites found in each category (see also electronic supplementary material, table S1). Tissue was processed using the colon peeling procedure (Methods). (*b*) Percentage EdU+ve parasites in infected cells during acute and chronic infections in relation to nest size. (*c*) Relating nest size to the % EdU+ve parasites during acute stage infection. Each point corresponds to a specific nest size (*x*-axis), and the corresponding %EdU+ve mean percentage value across all animals (*y*-axis). The *R*^2^ value was determined by linear regression.

Colon samples were excised 18 h after the second injection, and the incorporated EdU visualized (Methods). We observed significantly greater levels of parasite labelling in tissue obtained from acute stage mice than from those that were chronically infected (70% versus 20%) (figures 3 and 4; electronic supplementary material, figure S3). Therefore, during the acute stage, a greater fraction of the parasite population is replicating their nuclear and/or mitochondrial DNA at any specific time point. By inference, the amastigote replication rate must be slower during chronic infections, at least in this tissue location. There were no differences in the data derived from colonic tissue processed by the two differing methodologies (grey and yellow dots, figure 3*b*). We further observed that during the acute stage, there was a positive correlation between the number of parasites per infected cell and the percentage of parasites where DNA synthesis was ongoing (figure 4*a–c*). Large parasites nests were less common during the acute stage, with few instances where infected cells contained more than 50 parasites (figure 4). We did attempt to quantify, for comparative purposes, the relative level of EdU labelling in each parasite. However, since most images were taken with whole mounted tissue sections, the variable depths of parasites from the surface made this technically challenging. As judged by visual inspection, the majority of EdU+ve parasites in any one cell were labelled to a similar extent (figure 3*c–e*; electronic supplementary material, figure S3). Since smooth muscle cells, the most frequently infected cell type in the colon [29], are typically in a state of cell cycle arrest, the nuclei of host cells were generally unlabelled.

**Figure 5.**
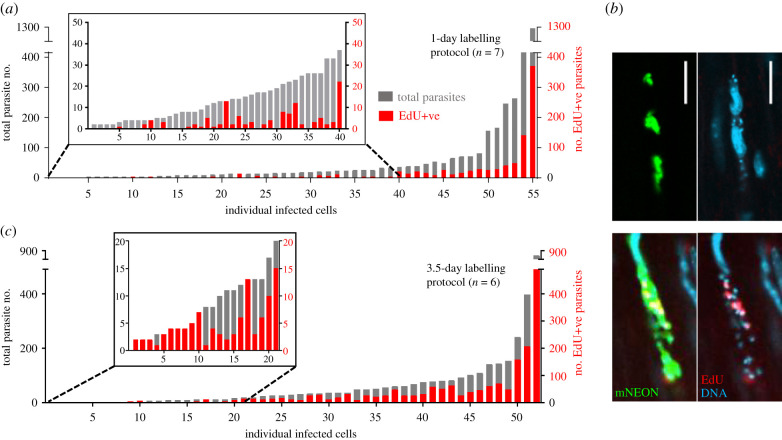
Increased chronic stage EdU incorporation by parasites with the 3.5-day protocol. (*a*) Level of EdU incorporation in the 55 infected cells detected in the whole mounted colonic gut walls from seven chronic stage mice treated with the 1-day labelling protocol. The total parasite content (grey) and the number that were EdU+ve (red) are indicated. (*b*) Upper images; an infected cell containing no EdU+ve parasites after labelling with the 1-day protocol. Lower images; an infected cell from the same colonic tissue that contained EdU+ve parasites (see also electronic supplementary material, figure S3 and table S1). Parasites, green. (*c*) EdU incorporation in 52 infected cells detected in six chronically infected mice treated with the 3.5-day labelling protocol. Every infected cell in the colonic gut walls of these mice contained at least one EdU+ve parasite.

In chronically infected mice, only a minority of parasites incorporated EdU when the 1-day protocol was used (figure 3; electronic supplementary material, figure S3), and in approximately 20% of infected cells, none of the parasites were labelled (figure 5*a,b*). A similar level of heterogeneous incorporation was observed in skeletal muscle, another site of parasite persistence in chronically infected C3H/HeN mice, and a tissue where parasites are often found in large nests (electronic supplementary material, figure S4, as example). When labelling was extended over 3.5 days (a total of seven injections) (figure 3*a*), there was a 2.3-fold increase in the number of labelled parasites, with approximately half of those in the colon being EdU+ve (figures 3*b* and 5*c*; electronic supplementary material, figure S3). With this more prolonged protocol, every infected host cell that we examined contained at least one labelled parasite (figure 5*c*). However, the percentage of EdU+ve parasites within the population was still significantly lower than during the acute stage, when the 1-day labelling protocol was used (figure 3). In combination, these data indicate that during chronic infection of the colon, there is a general reduction in the number of parasites in S-phase. As judged by bioluminescence *ex vivo* imaging of organs and tissues, neither the 1-day nor the 3.5-day EdU injection protocols had any detectable effect on the levels of infection or on tissue-specific parasite dissemination (electronic supplementary material, figure S5).

### 3.4. Long-term infection of individual colonic smooth muscle cells is not common during the chronic stage

**Figure 6.**
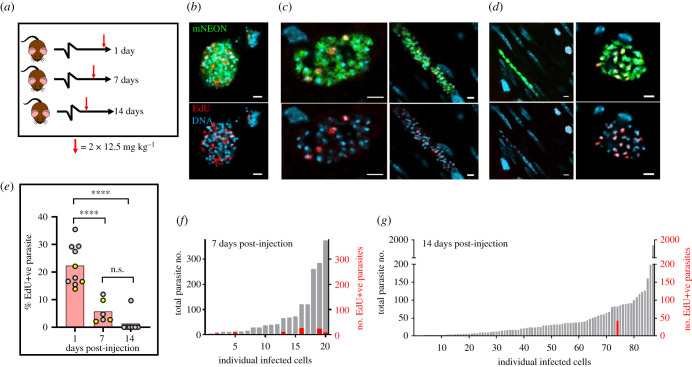
EdU labelling experiments reveal that parasite occupation of colonic host cells during chronic stage infections is not long term. (*a*) Schematic of the labelling protocol. EdU was injected in two 12.5 mg kg^−1^ doses, 6 h apart (as in figure 3*a*). After 1, 7 or 14 days, mice were sacrificed, colonic tissue excised, and infected cells detected by *ex vivo* imaging and confocal microscopy (Methods). (*b*) Representative images of parasite EdU incorporation following the 1-day labelling protocol (see also figure 3*d*). (*c*) EdU incorporation assessed 7 days post-injection. (*d*) Left-hand image; EdU incorporation assessed 14 days post-injection. Typically, infected cells contained no EdU+ve parasites. Right-hand image; the single example of an infected host containing EdU+ve parasites after an exhaustive search of colon mounts from eight mice. Scale bars, 20 µm. (*e*) Mean % EdU+ve parasites found in infected colonic cells. Each data point represents a single mouse; 1-day post-injection, *n* = 10; 7-day post-injection, *n* = 6; 14-day post-injection, *n* = 8. The total number of parasites detected, imaged and designated as EdU+ve or EdU−ve in each mouse varied from 47 to 2468, with an average of 608. The yellow data points indicate colons processed by standard histological sectioning, and grey data points highlight those processed through peeling away of the mucosal layer and whole mounting of the remaining colonic gut wall (Methods). Statistical analysis of treatment conditions was performed as described (Methods); ****p ≤ 0.0001. There was no significant difference (n.s.) between the 7- and 14-day post-injection groups. (*f*) Percentage parasites that were EdU+ve in infected colonic cells from mice sacrificed 7 days post-injection (*n* = 3). Grey bar indicates the total parasite number in each infected cell; red bar indicates the percentage EdU+ve. (*g*) Similar analysis of EdU positivity in infected cells found in mice 14 days post-injection (*n* = 7).

To further investigate parasite replication during chronic stage infections, we undertook experiments to assess the extent and stability of parasite labelling 7 and 14 days after EdU injection using the 1-day labelling protocol (figure 6*a*). When mice were examined by *ex vivo* bioluminescence imaging after 14 days, there had been no measurable impact on the parasite burden or tissue distribution (electronic supplementary material, figure S5). At 7-day post-injection point, EdU+ve parasites were still readily detectable, although there was a fourfold decrease in their relative abundance within the population, and only approximately 40% of infected cells contained any labelled parasites (figure 6*b,c,e,f*). By 14 days post-injection, out of the 87 infected cells detected in the colons of eight mice, just one contained EdU+ve amastigotes (figure 6*d,e,g*). Using serial Z-stacking, we established that this infected cell contained 82 parasites, 42 of which were labelled. Given this profile, the most likely explanation is that this host cell had remained infected for at least 14 days, with the parasites in a state of low proliferation. The EdU+ve parasites cannot have undergone many replication cycles during this period, since the labelling intensity was similar to that in parasites examined 1-day post-injection. Furthermore, in dividing cells, incorporated nucleosides become undetectable after two to five generations, assuming random segregation of daughter chromosomes [42,43]. It can be further inferred from the rarity of cells containing EdU+ve parasites (figure 6*d,g*), that long-term occupancy of individual colonic smooth muscle cells by *T. cruzi* is not a common feature of chronic stage infections, even though this tissue is a site of parasite persistence. In the vast majority of cases, therefore, the normal infection cycle of parasite replication, host cell lysis and re-infection appears to continue during the chronic stage, albeit at a reduced rate. Finally, the observation of multiple labelled amastigotes within a single host cell 14 days after injection (figure 6*d*) demonstrates that EdU is stable once it has been incorporated into the *T. cruzi* genome, and that it is not readily susceptible to removal by metabolic or DNA repair pathways. This stability has similarities with the situation in mice, where Merkel cells labelled during pregnancy remained EdU+ve in offspring nine months after birth [44].

## 4. Discussion

The report that *T. cruzi* can undergo a form of spontaneous dormancy has highlighted the possibility that the proliferation status of the parasite could have a role in long-term persistence and contribute to the high rate of treatment failure [13]. Improvements in tissue processing and imaging procedures [29] have allowed us to explore this further by providing a platform to investigate parasite replication in the colon of chronically infected mice, a tissue that supports long-term *T. cruzi* persistence at extremely low levels [27]. Our major finding is that during chronic infections, the proportion of intracellular parasites in S-phase is significantly lower than it is during the acute stage. In acute infections, 70% of parasites were EdU+ve after using the 1-day protocol, compared with 20% during the chronic stage (figures 3 and 4; electronic supplementary material, figure S3). This is unlikely to reflect reduced EdU uptake or bioavailability during chronic infections, as the staining intensity of individual EdU+ve parasites was similar during both stages of the disease, with the only apparent difference being the proportion of amastigotes that were positive. The most parsimonious explanation is that *T. cruzi* amastigotes proliferate at a slower rate in the chronic stage, at least in this tissue location, and were, therefore, less likely to be replicating their DNA during the period of EdU exposure. It is implicit from this that *T. cruzi* amastigotes have the capability of responding to environmental signals that are specific to chronic and/or acute stage disease, such as nutrient availability or indicators of the immune response. In the latter case, the observation that immunosuppression rapidly reactivates the infection [30], suggests that the host response could be a driver for slow replication, either directly or indirectly. These observations are in line with previous reports that amastigote replication and cell cycle kinetics can be subject to reversible stress-induced inhibition *in vitro* [26]. A response mechanism of this type could also account for the correlation between amastigote growth rate and nest size during acute infections (figure 4).

**Figure 7.**
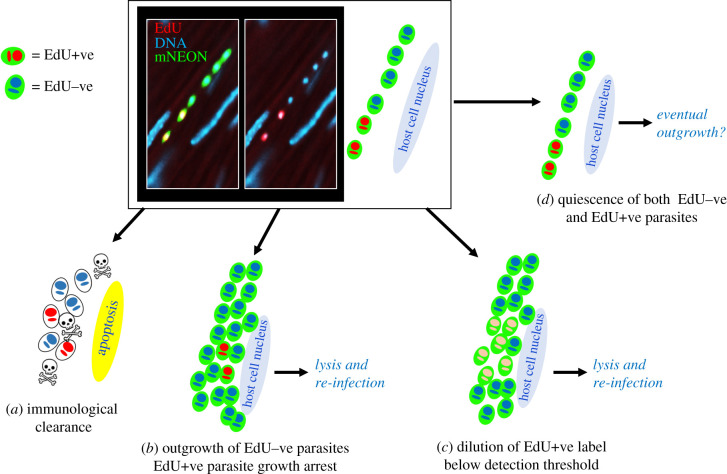
Schematic highlighting the possible fates of host cells and parasites following EdU labelling. The image shows a colon smooth muscle section from a chronically infected mouse following treatment with the 1-day labelling protocol (figure 3). EdU labelling (red) appears as yellow against the green background of parasite fluorescence. (*a*) Following EdU exposure, the infected cell and parasites could be cleared by the host immune response. (*b*) There could be outgrowth of EdU−ve parasites in an infected cell. The EdU+ve subset, which would have been in S-phase during exposure, might enter cell cycle arrest after incorporation. (*c*) If EdU incorporation is below the toxicity threshold for cell cycle arrest, amastigote proliferation will lead to serial dilution of the label. (*d*) After EdU incorporation by a subset of parasites, all the amastigotes in the cell might enter a slow proliferative state. This appears to be a rare event (figure 6*d,g*).

The heterogeneous nature of labelling during the chronic stage, with many parasites being EdU−ve (figure 3; electronic supplementary material, figure S3), should not be interpreted as being indicative of spontaneous dormancy. Rather, it provides further evidence that parasite replication within individual host cells is asynchronous [31]. The observation that some intracellular parasites do not incorporate EdU reflects that amastigotes exist in a range of replicative states within individual infected cells, an inference supported by the cumulative nature of EdU labelling. There are several possible fates for parasites labelled with EdU during chronic stage infections, as outlined in figure 7. Given our data, which show a steady reduction in the percentage of EdU+ve parasites per infected cell over time (figure 6), continued growth of these amastigotes and dilution of the label below the level of detection (figure 7*c*) would appear to be the most likely outcome.

Although incorporation of EdU into replicating DNA is widely used in proliferation studies, it can lead to a DNA damage response and cell cycle arrest [36–38]. With *T. cruzi,* the measurable effect of EdU toxicity is time-dependent [40], and here, we showed that 6 h exposure *in vitro* at 1–2 µM is sufficient to inhibit amastigote replication (figure 2*d*). Therefore, studies on *T. cruzi* proliferation, dormancy and persistence could be confounded if EdU exposure is continuous. In the case of *in vivo* administration, EdU toxicity should be less problematic, because of the short clearance time of thymidine analogues [41]. Consistent with this, we found no detectable impact of EdU exposure on parasite burden or tissue tropism in chronically infected mice (electronic supplementary material, figure S5). In these infection experiments, where incorporation was employed as an endpoint assay, providing snapshots of DNA replication within the parasite population, EdU toxicity would not be expected to compromise the outcome. This contrasts with *in vitro* experiments where EdU exposure can be continuous, resulting in inter-strand cross-linking and double-strand breaks, which trigger DNA damage signalling and cell cycle arrest [37]. This is a ubiquitous response in all cells with DNA damage sensing machinery [45,46]. Spontaneous dormancy has been proposed as a mechanism that could account for parasite persistence after therapy [13,47]. However, in *T. cruzi,* the front-line drug benznidazole can cause mutagenesis, disruption to DNA repair pathways and chromosome instability [48,49]. Therefore, an alternative explanation could be that benznidazole-induced DNA damage responses trigger cell cycle arrest and a transient dormant-like state, which protects some parasites from further drug-induced toxicity, ultimately leading to relapse after the successful completion of DNA repair.

Our findings do not exclude the possibility that some parasites might have the potential to enter a canonical dormant state at specific points in the life cycle. However, they more strongly suggest that rather than being a discrete biological stage, a dormancy-like phenotype in *T. cruzi* might be better described as representing one end of the normal proliferation spectrum. The cell cycle plasticity necessary for this has already been reported in amastigotes [26]. Therefore, the reduced rate of *T. cruzi* replication during the chronic stage could be a phenomenon more analogous to the biochemical quiescence and reduced proliferation exhibited by *Leishmania* [19,20], than to the more definitive dormant state displayed by *T. gondii* and some *Plasmodium* species [3]. Resolving this question and understanding the mechanisms involved has particular importance for Chagas disease drug development strategies. As described in this paper (figures 1 and 2), there are limitations to the cell tracker dye and DNA labelling methodologies that have previously been applied to investigate *T. cruzi* proliferation and quiescence. Therefore, new approaches are urgently required.

## Supplementary Material

Figures S1 - S5

## Supplementary Material

Table S1

## Supplementary Material

Video S1

## Data accessibility

All cell lines described in this paper are freely available and can be obtained by contacting the corresponding author (J.M.K.).
