# Supplementary material for: Trypanosoma cruzi amastigotes that persist in the colon during chronic stage murine infections have a reduced replication rate
Source: Open Biol. 2020 Dec 16;10(12):200261. doi: 10.1098/rsob.200261 (PMC7776577; doi:10.1098/rsob.200261)
Supplement: Figures S1 - S5 [file rsob200261supp1.pptx]

## Slide 1
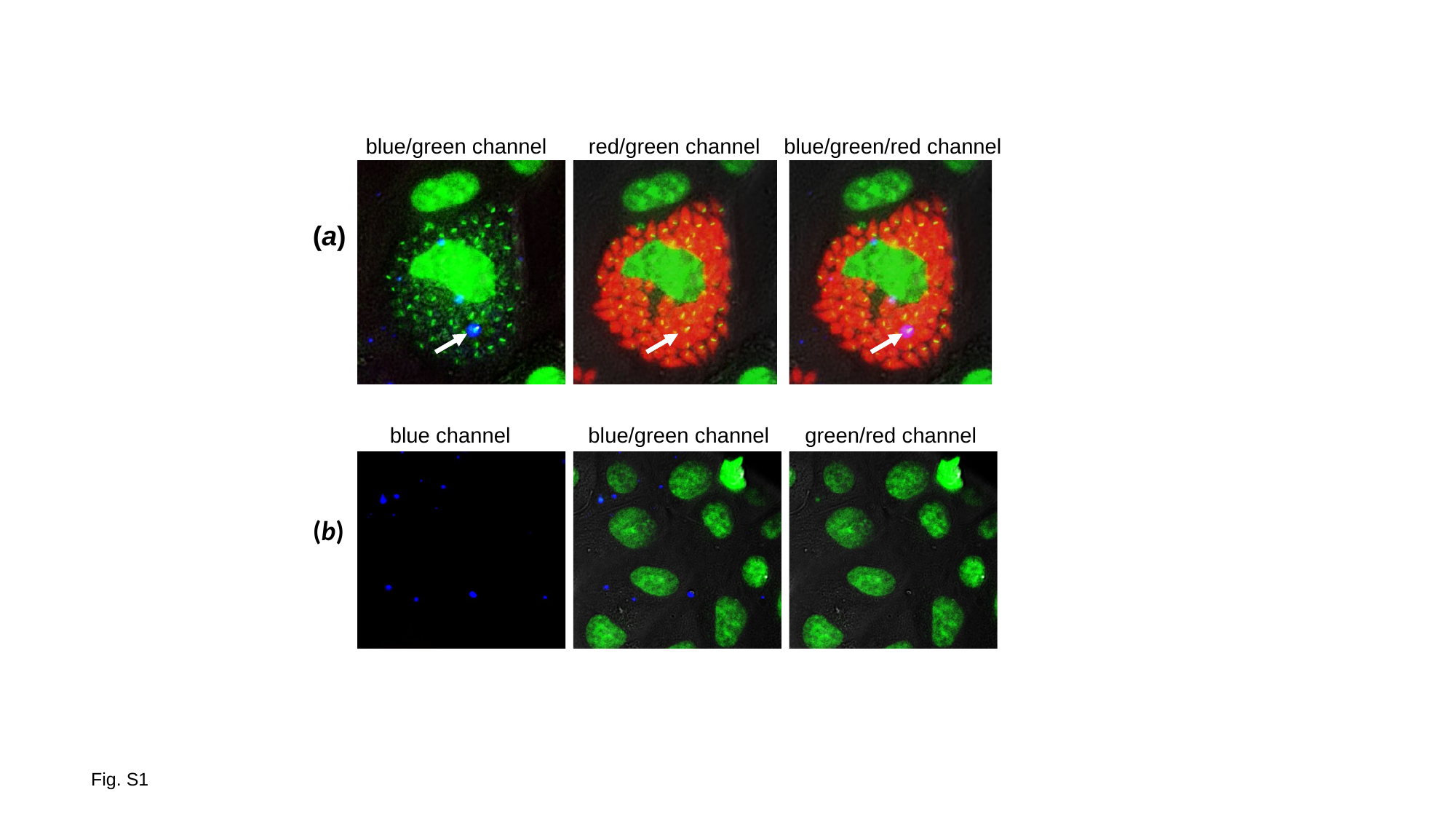

blue/green channel red/green channel blue/green/red channel
(a)
(b)
blue channel blue/green channel green/red channel
Fig. S1

## Slide 2
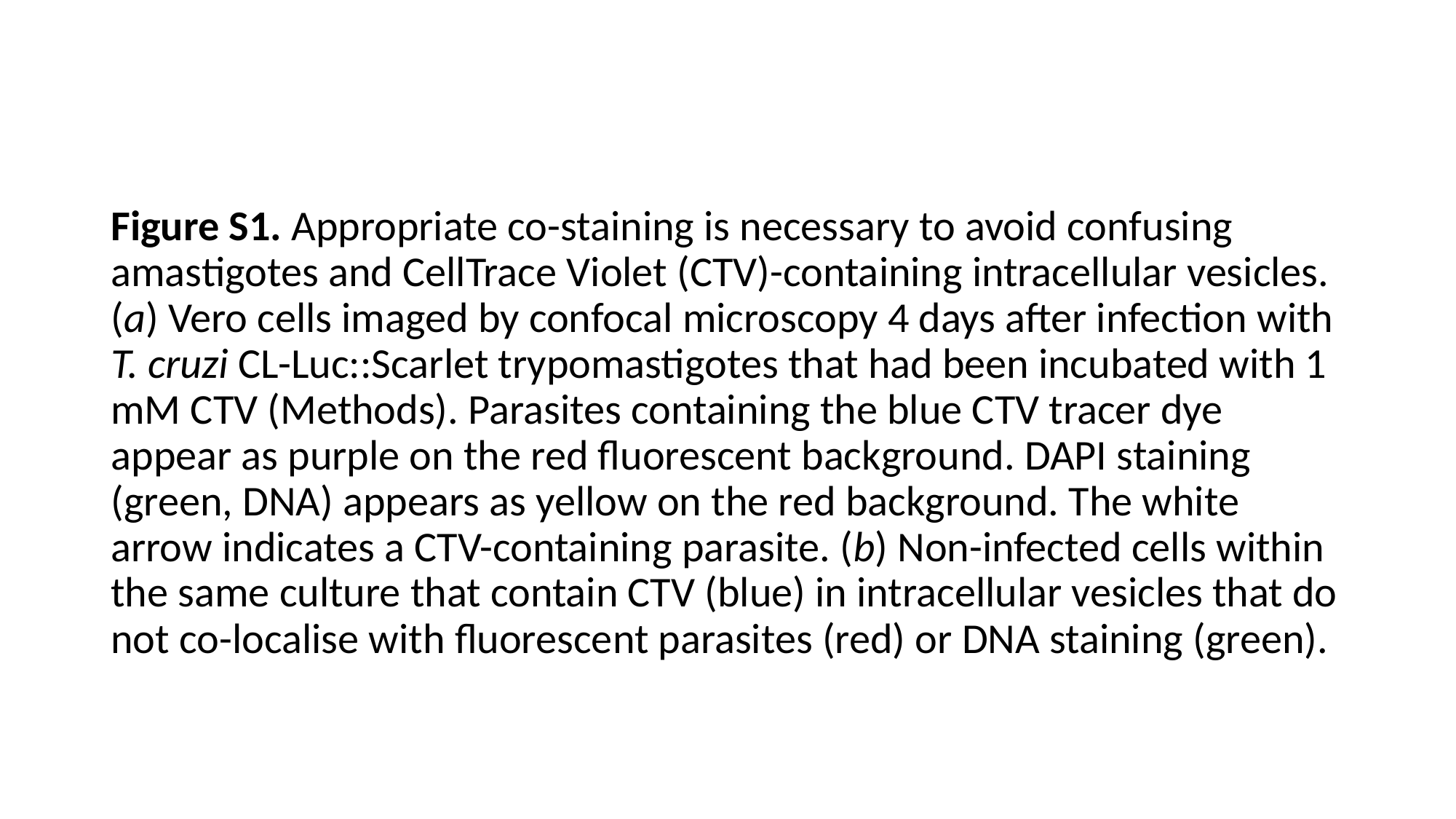

Figure S1. Appropriate co-staining is necessary to avoid confusing amastigotes and CellTrace Violet (CTV)-containing intracellular vesicles. (a) Vero cells imaged by confocal microscopy 4 days after infection with T. cruzi CL-Luc::Scarlet trypomastigotes that had been incubated with 1 mM CTV (Methods). Parasites containing the blue CTV tracer dye appear as purple on the red fluorescent background. DAPI staining (green, DNA) appears as yellow on the red background. The white arrow indicates a CTV-containing parasite. (b) Non-infected cells within the same culture that contain CTV (blue) in intracellular vesicles that do not co-localise with fluorescent parasites (red) or DNA staining (green).

## Slide 3
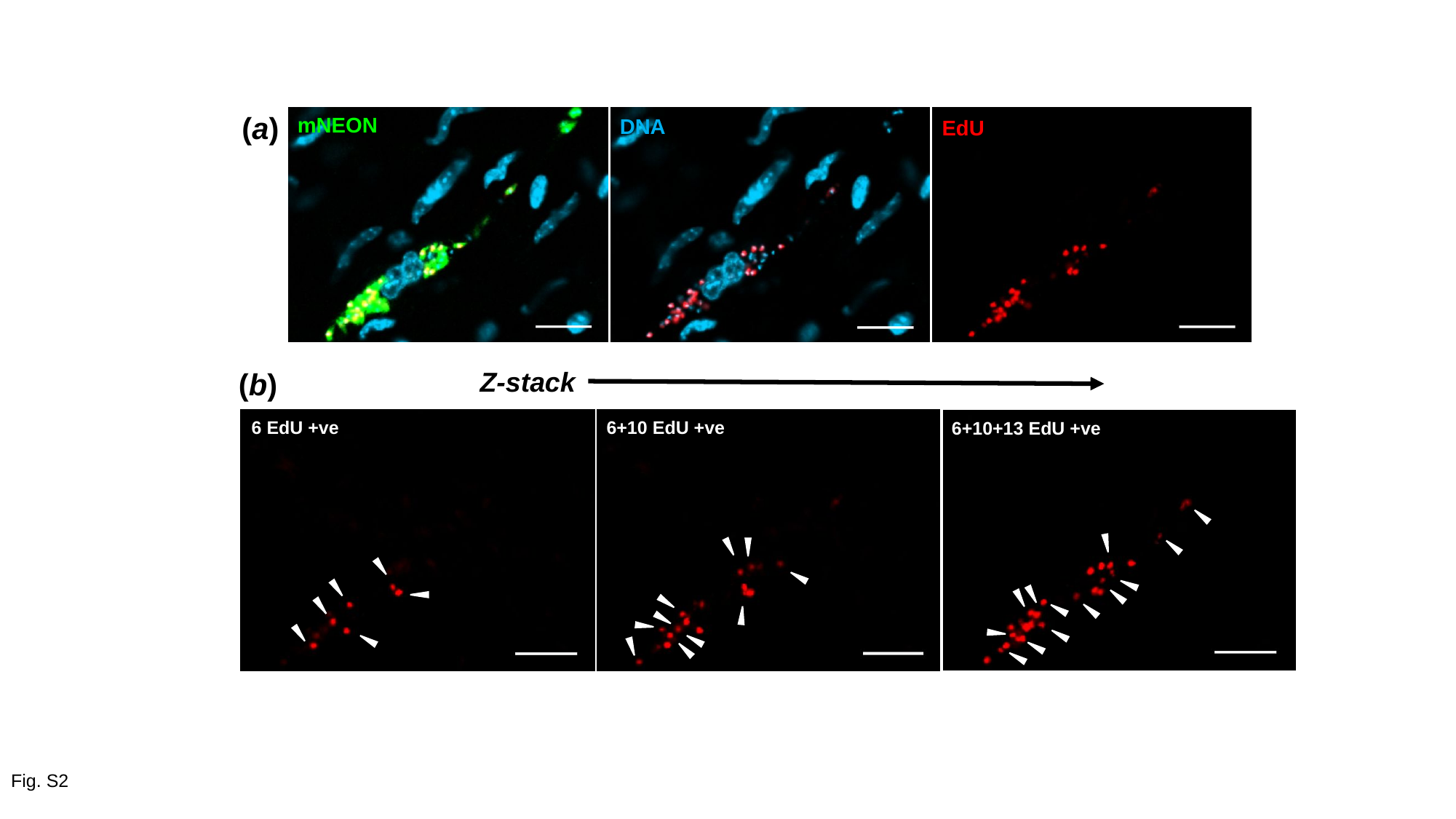

(a)
mNEON
DNA
EdU
Z-stack
(b)
6+10 EdU +ve
6 EdU +ve
6+10+13 EdU +ve
Fig. S2

## Slide 4
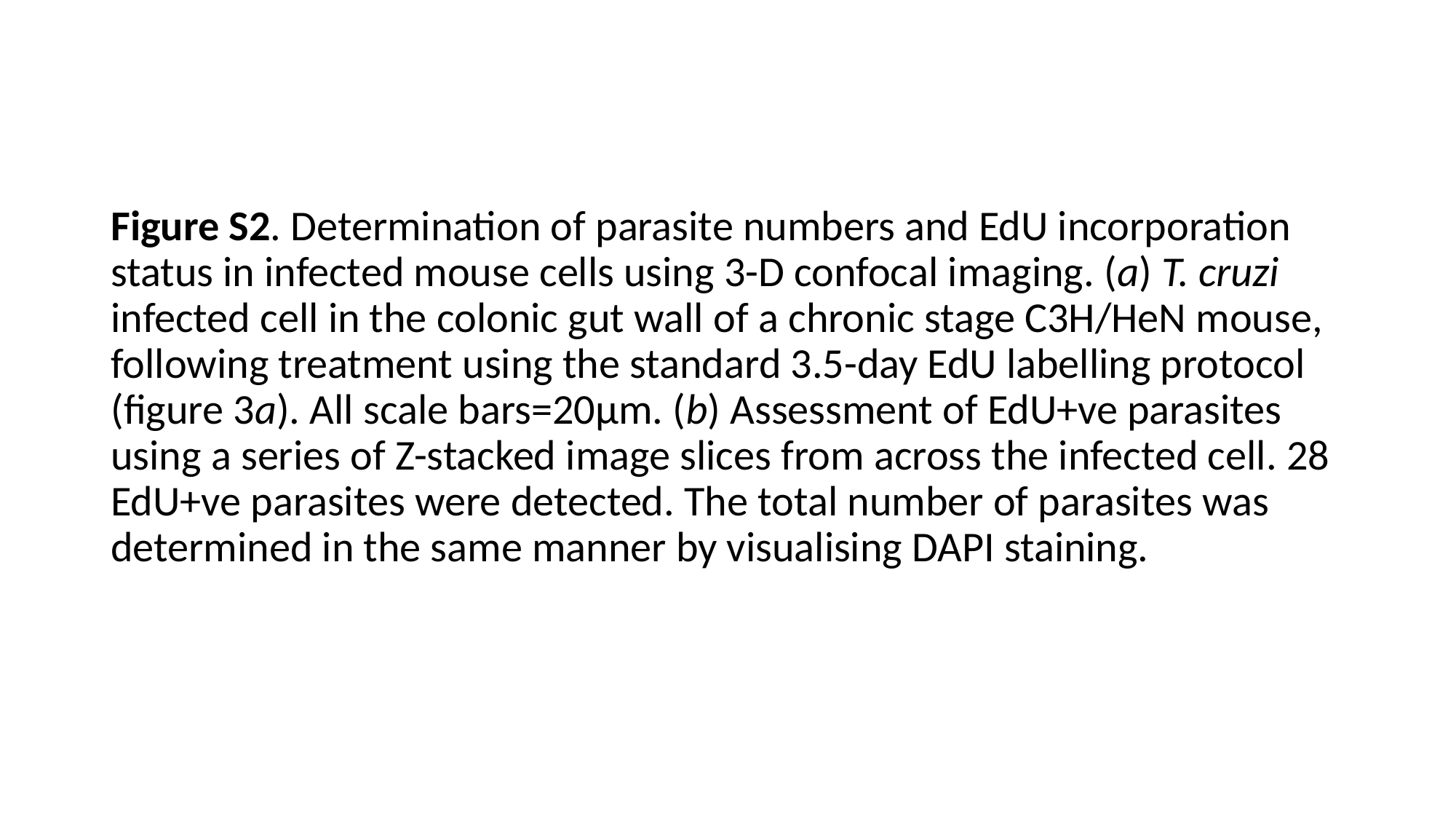

Figure S2. Determination of parasite numbers and EdU incorporation status in infected mouse cells using 3-D confocal imaging. (a) T. cruzi infected cell in the colonic gut wall of a chronic stage C3H/HeN mouse, following treatment using the standard 3.5-day EdU labelling protocol (figure 3a). All scale bars=20μm. (b) Assessment of EdU+ve parasites using a series of Z-stacked image slices from across the infected cell. 28 EdU+ve parasites were detected. The total number of parasites was determined in the same manner by visualising DAPI staining.

## Slide 5
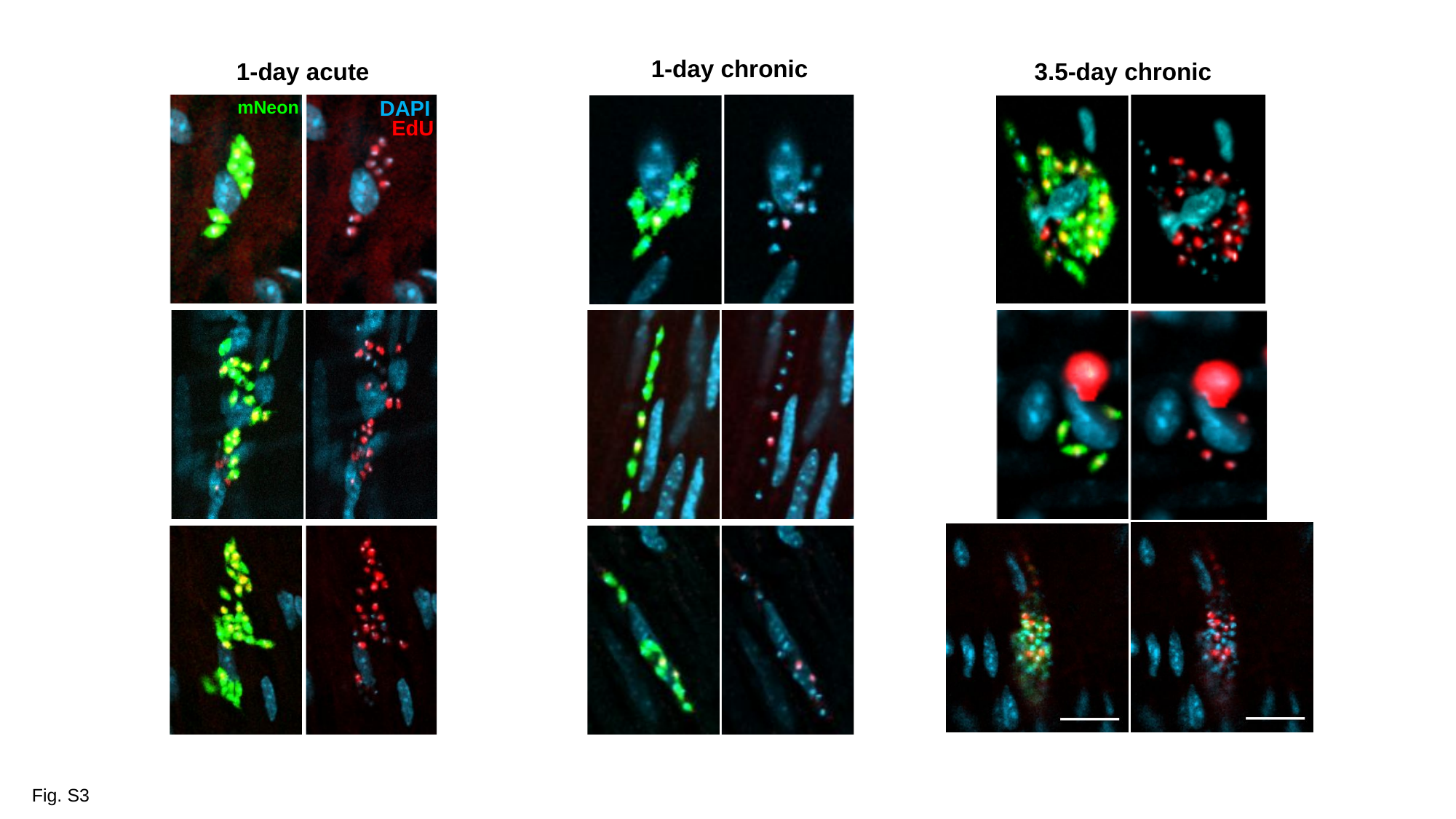

1-day chronic
1-day acute
3.5-day chronic
mNeon
DAPI
 EdU
Fig. S3

## Slide 6
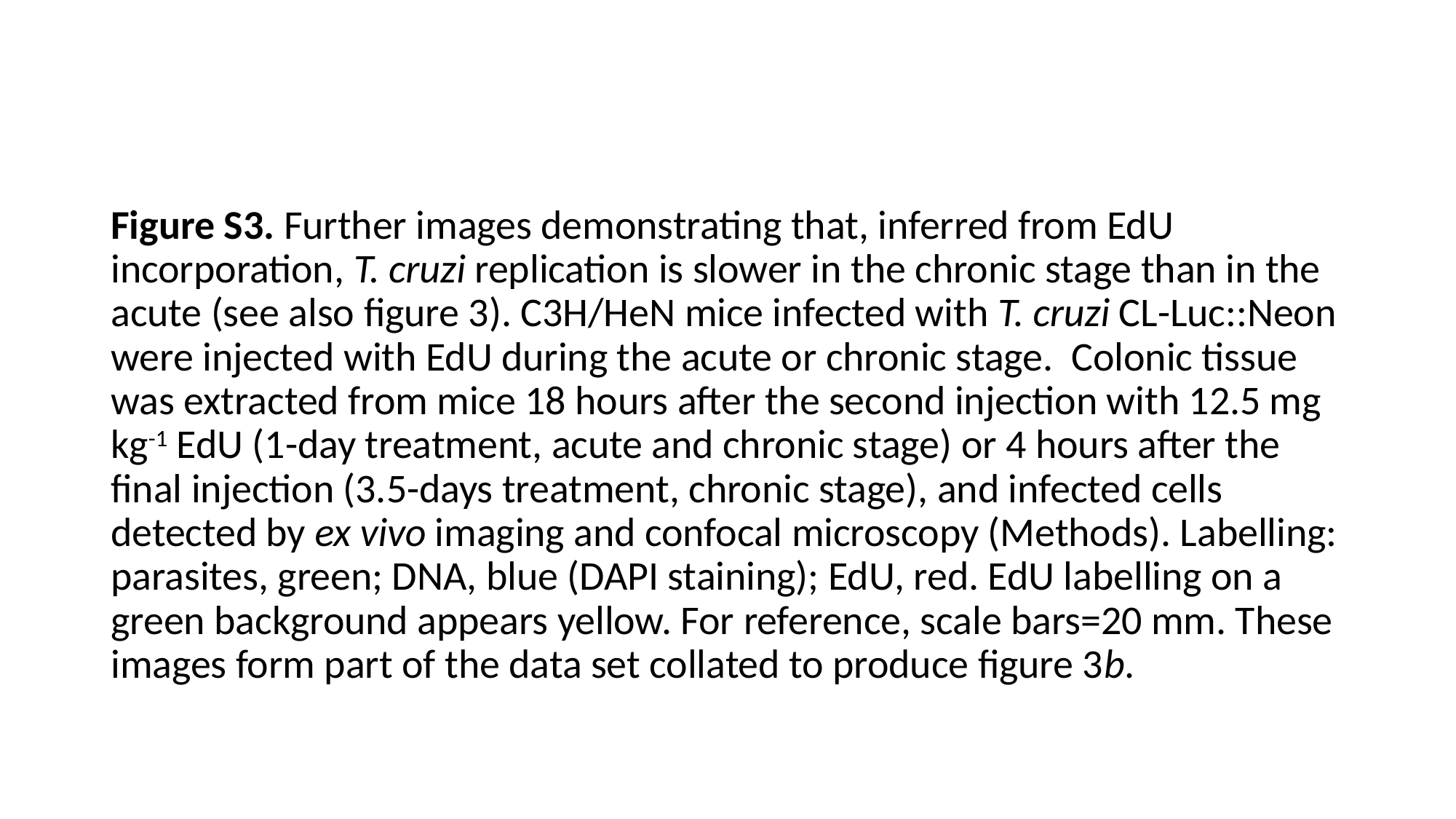

Figure S3. Further images demonstrating that, inferred from EdU incorporation, T. cruzi replication is slower in the chronic stage than in the acute (see also figure 3). C3H/HeN mice infected with T. cruzi CL-Luc::Neon were injected with EdU during the acute or chronic stage. Colonic tissue was extracted from mice 18 hours after the second injection with 12.5 mg kg-1 EdU (1-day treatment, acute and chronic stage) or 4 hours after the final injection (3.5-days treatment, chronic stage), and infected cells detected by ex vivo imaging and confocal microscopy (Methods). Labelling: parasites, green; DNA, blue (DAPI staining); EdU, red. EdU labelling on a green background appears yellow. For reference, scale bars=20 mm. These images form part of the data set collated to produce figure 3b.

## Slide 7
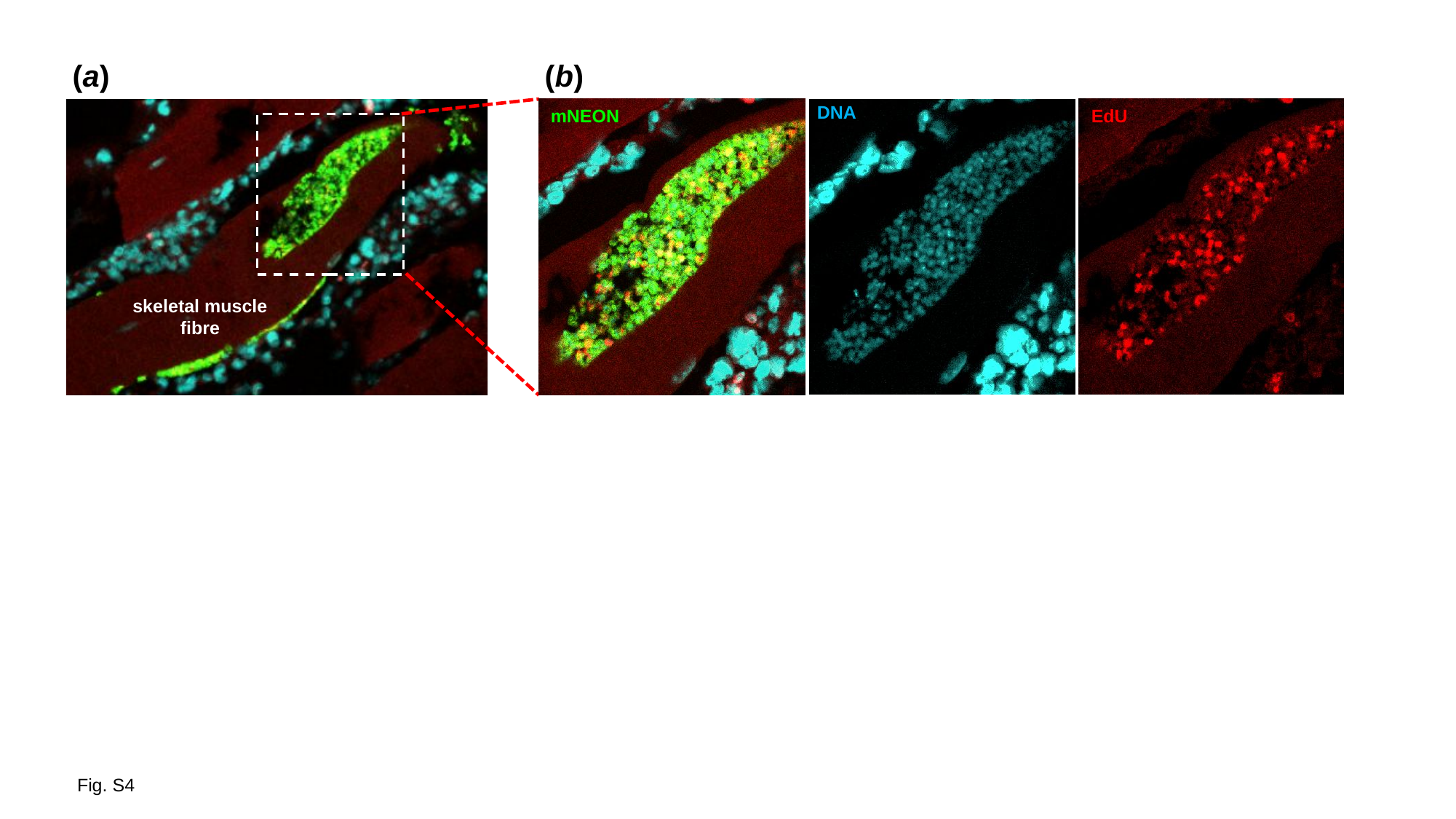

(a) (b)
DNA
mNEON
EdU
skeletal muscle fibre
Fig. S4

## Slide 8
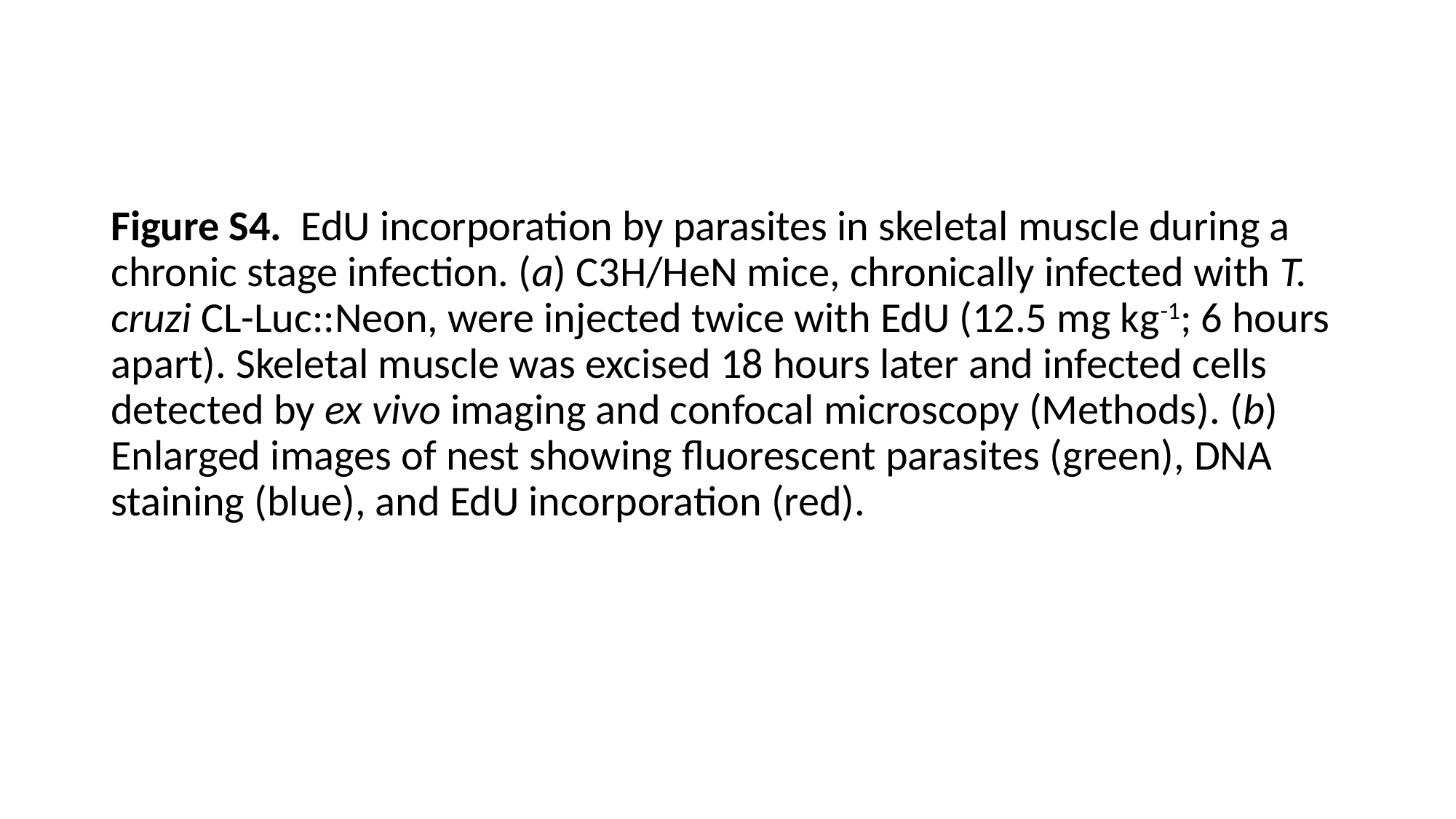

Figure S4. EdU incorporation by parasites in skeletal muscle during a chronic stage infection. (a) C3H/HeN mice, chronically infected with T. cruzi CL-Luc::Neon, were injected twice with EdU (12.5 mg kg-1; 6 hours apart). Skeletal muscle was excised 18 hours later and infected cells detected by ex vivo imaging and confocal microscopy (Methods). (b) Enlarged images of nest showing fluorescent parasites (green), DNA staining (blue), and EdU incorporation (red).

## Slide 9
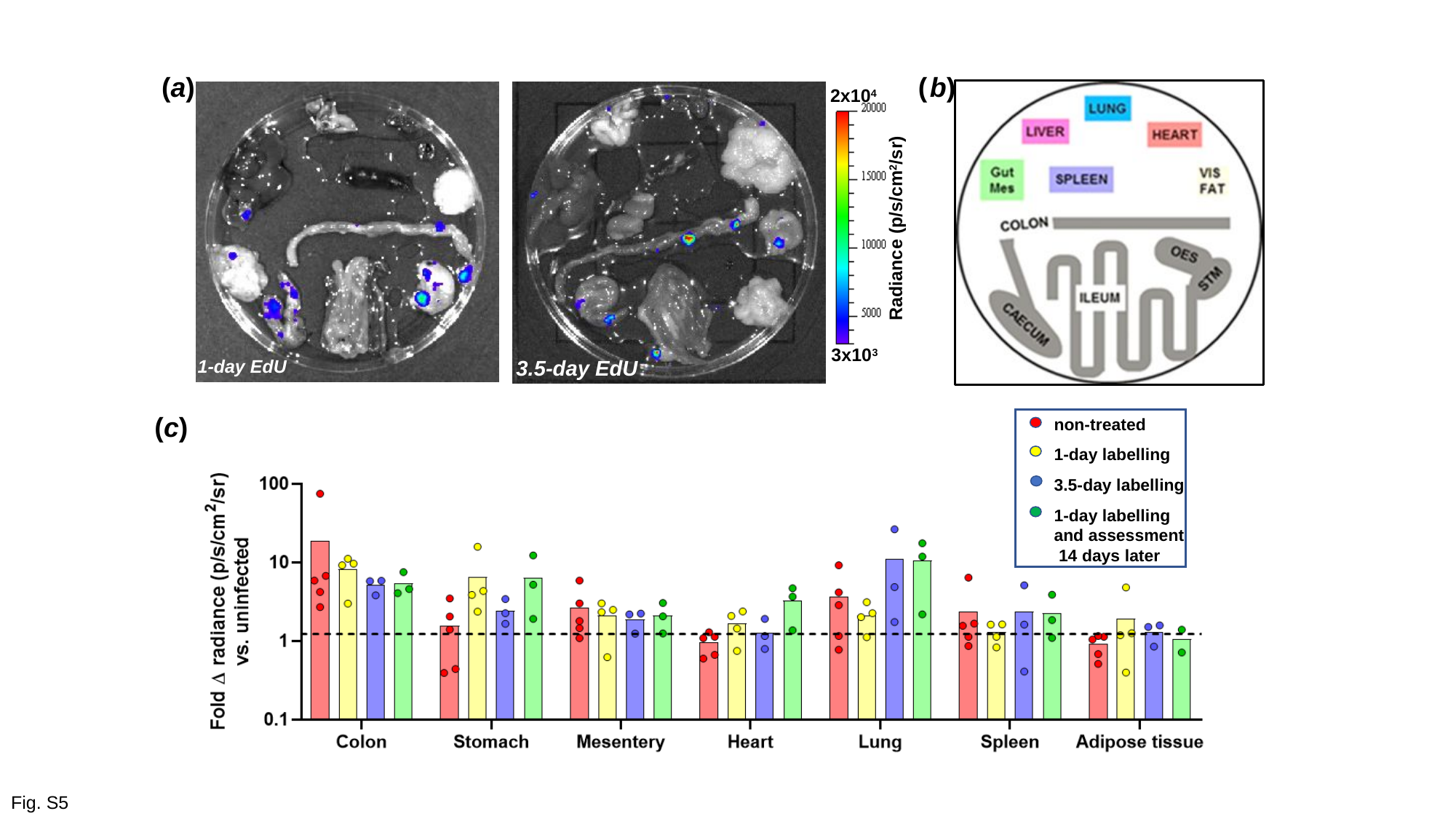

(a) (b)
2x10^4^
Radiance (p/s/cm2/sr)
3x10^3^
3.5-day EdU
1-day EdU
non-treated
1-day labelling
3.5-day labelling
1-day labelling
and assessment
 14 days later
(c)
Fig. S5

## Slide 10
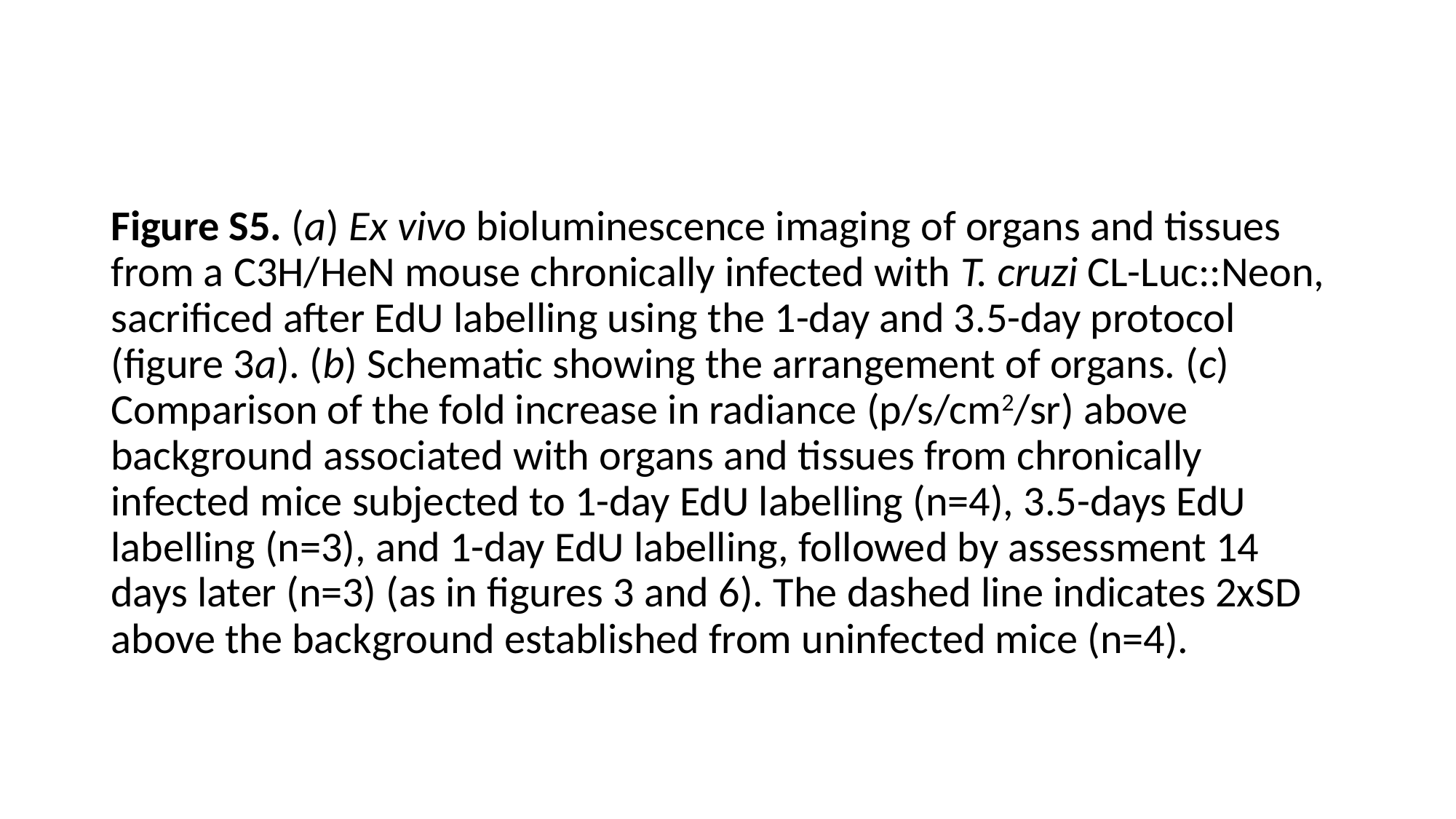

Figure S5. (a) Ex vivo bioluminescence imaging of organs and tissues from a C3H/HeN mouse chronically infected with T. cruzi CL-Luc::Neon, sacrificed after EdU labelling using the 1-day and 3.5-day protocol (figure 3a). (b) Schematic showing the arrangement of organs. (c) Comparison of the fold increase in radiance (p/s/cm2/sr) above background associated with organs and tissues from chronically infected mice subjected to 1-day EdU labelling (n=4), 3.5-days EdU labelling (n=3), and 1-day EdU labelling, followed by assessment 14 days later (n=3) (as in figures 3 and 6). The dashed line indicates 2xSD above the background established from uninfected mice (n=4).
